# Rabies encephalitis in a preschool child following postexposure prophylaxis

**DOI:** 10.1371/journal.pntd.0009045

**Published:** 2021-02-18

**Authors:** K. M. Chaitra, Sandeep Ballal, N. R. Ramesh Masthi, D. H. Ashwath Narayana, H. T. Yashodha, Mansi Kumar, Afroza Asiya, Divya Bharathi G., Sanjay S. C.

**Affiliations:** 1 Department of Paediatrics Kempegowda Institute of Medical Sciences, Bengaluru, Karnataka, India; 2 Department of Radiology, Kempegowda Institute of Medical Sciences, Bengaluru, Karnataka, India; 3 Department of Community Medicine, Kempegowda Institute of Medical Sciences, Bengaluru, Karnataka, India; University of Iowa, UNITED STATES

## Abstract

We report a case of rabies encephalitis in a 4½-year-old male child with an exposure to a suspect rabid dog. The child developed rabies 25 days after receiving postexposure prophylaxis. Rabies immunoglobulin (RIG) is currently administered according to body weight. In high-risk exposures over the head and neck, local administration of RIG over and above the body weight depending on the site, size, and severity of exposure may help to prevent rabies death. There is a need for further studies to generate new evidence in this regard.

## Introduction

Rabies is an acute encephalitis caused by lyssavirus infection [1]. Rabies encephalitis has the highest fatality rate among infectious diseases with the average time interval from clinical disease onset to death reported to be 5 to 7 days in furious rabies and 11 days in paralytic rabies [2]. This case report is of a 4½ years old male child who developed rabies 25 days after exposure to a suspect rabid dog, despite having received rabies immunoglobulin (equine rabies immunoglobulin) into the wound within 4 hours of exposure and 4 doses of anti-rabies vaccine (ARV) according to the schedule (Essen regimen).

### Case presentation

A 4½ years old male child from a rural area near Kunigal taluk, Tumkur district (about 80 kms away from Bengaluru), Karnataka, India was admitted to the paediatric department of the medical college hospital with complaints of high-grade fever, liquids coming through the nose with feeding, dysphagia since 2 days, and irritability since 1 day.

**Fig 1:**
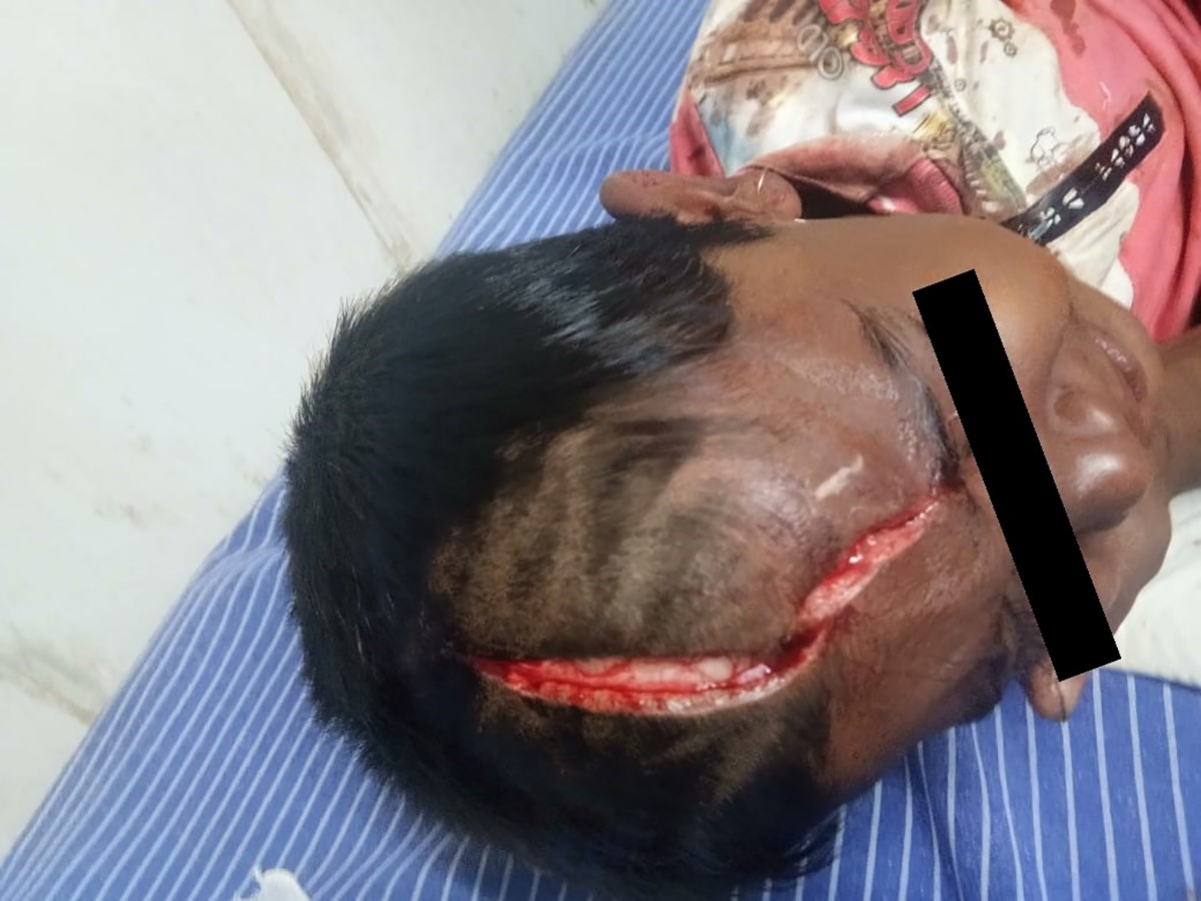
Category-III wound on the head.

The informants were parents of the child who gave history of the child being attacked by a suspected rabid dog 27 days previous to the admission. The dog had bitten other people on the same day, and it was killed with a suspicion of being rabid. A lacerated wound (WHO category-III) measuring about 15 cm in length and 2 cm in width extended from the forehead to middle of the head **(Fig 1).** The child received first aid (wound wash) and treatment (tetanus toxoid injection and intramuscular ARV (Abhayrab) (deltoid) at a local hospital. He was then referred to a public tertiary care hospital in the state capital for administration of rabies immunoglobulin (RIG) on the same day (within 4 hours). The wound was infiltrated with 2.7 ml (20 kg × 40 IU = 800 IU = 2.7 ml) of equine rabies immunoglobulin (EQUIRAB) diluted with normal saline in the ratio of 1:5 and sutured next day. Child was administrated 4 doses of ARV, i.e., on days 0 and 14, intramuscular (IM) route (local hospital, Kunigal), and days 3 and 7, intradermal (ID) route (public tertiary care hospital, Bengaluru).

On examination, the child was drowsy and irritable with heart rate of 126 beats/min, respiratory rate of 28 cycles/min, capillary filling time <3 s, and temperature 102°F. On CNS examination, the GCS score was E3 V4 M6. No classical signs of rabies like hydrophobia, aerophobia, or photophobia was observed.

On admission, the laboratory investigations showed haemoglobin 8.8 g/dl; total count 8.4 cells/μl; differential count: neutrophils 63%, lymphocytes 31%, and monocytes 6%; ESR 20 mm/h; platelet 305 platelets/μl; peripheral smear showed microcytic hypochromic anaemia; urea 12.49 mmol/L; creatine 30.50 μmol/L; uric acid 0.20 mmol/L; liver function test: serum bilirubin 8.55 μmol/L, SGOT 33 U/l, SGPT 7 U/l, ALP 7 U/l.

The child was started on antibiotics—injection ceftriaxone 1 g intravenous (IV) 12 hourly, injection paracetamol 20 ml (IV) 6 hourly, and injection mannitol 100 ml (IV) 8 hourly for symptomatic management. Fifth dose (day 28) ARV was administered in the hospital.

**Fig 2:**
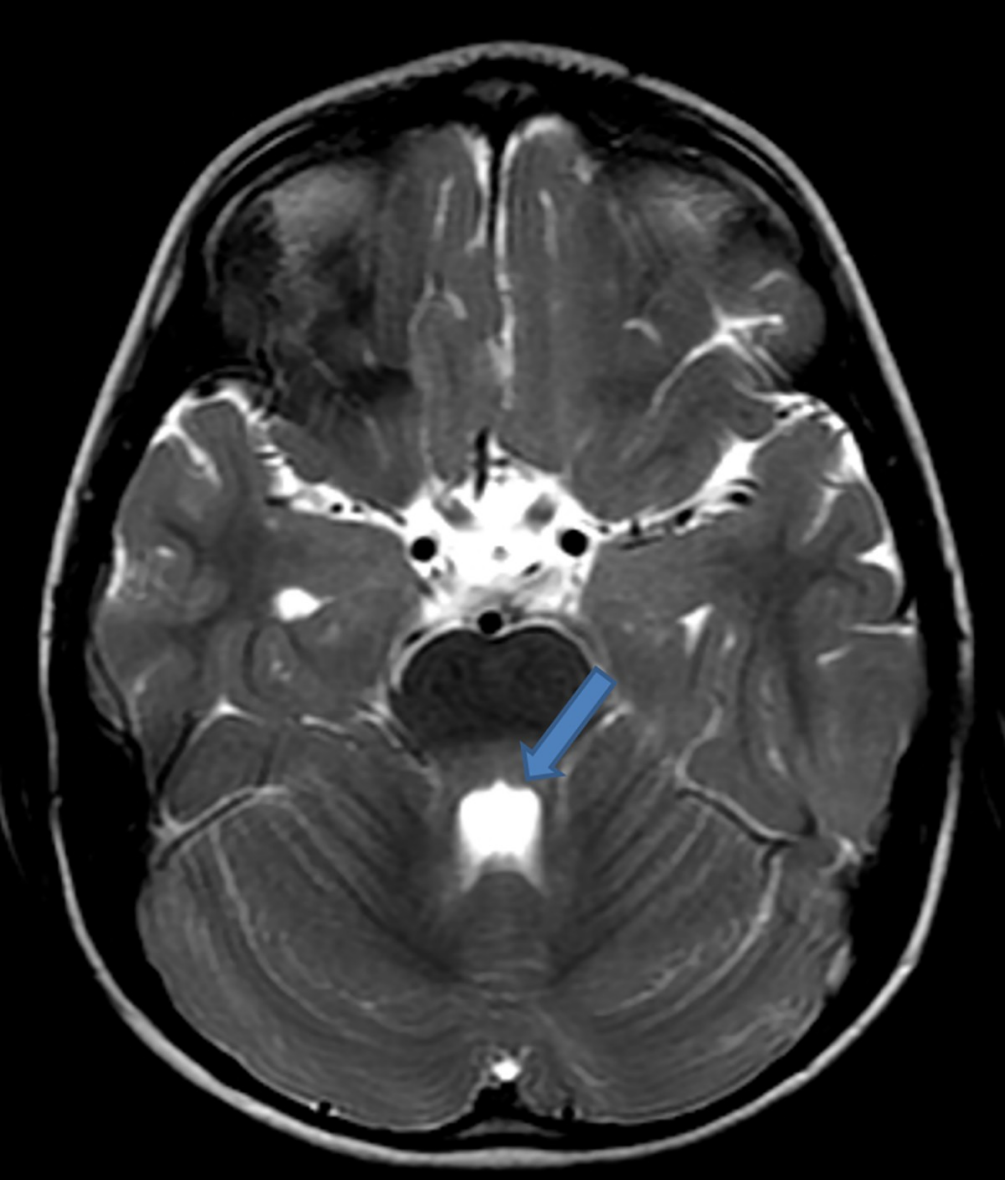
T2 hyperintensities noted in posterior 1/3 of pons. Pontomedullary junction, medulla oblongata, and posterior part of left temporal gyri.

**Fig 3:**
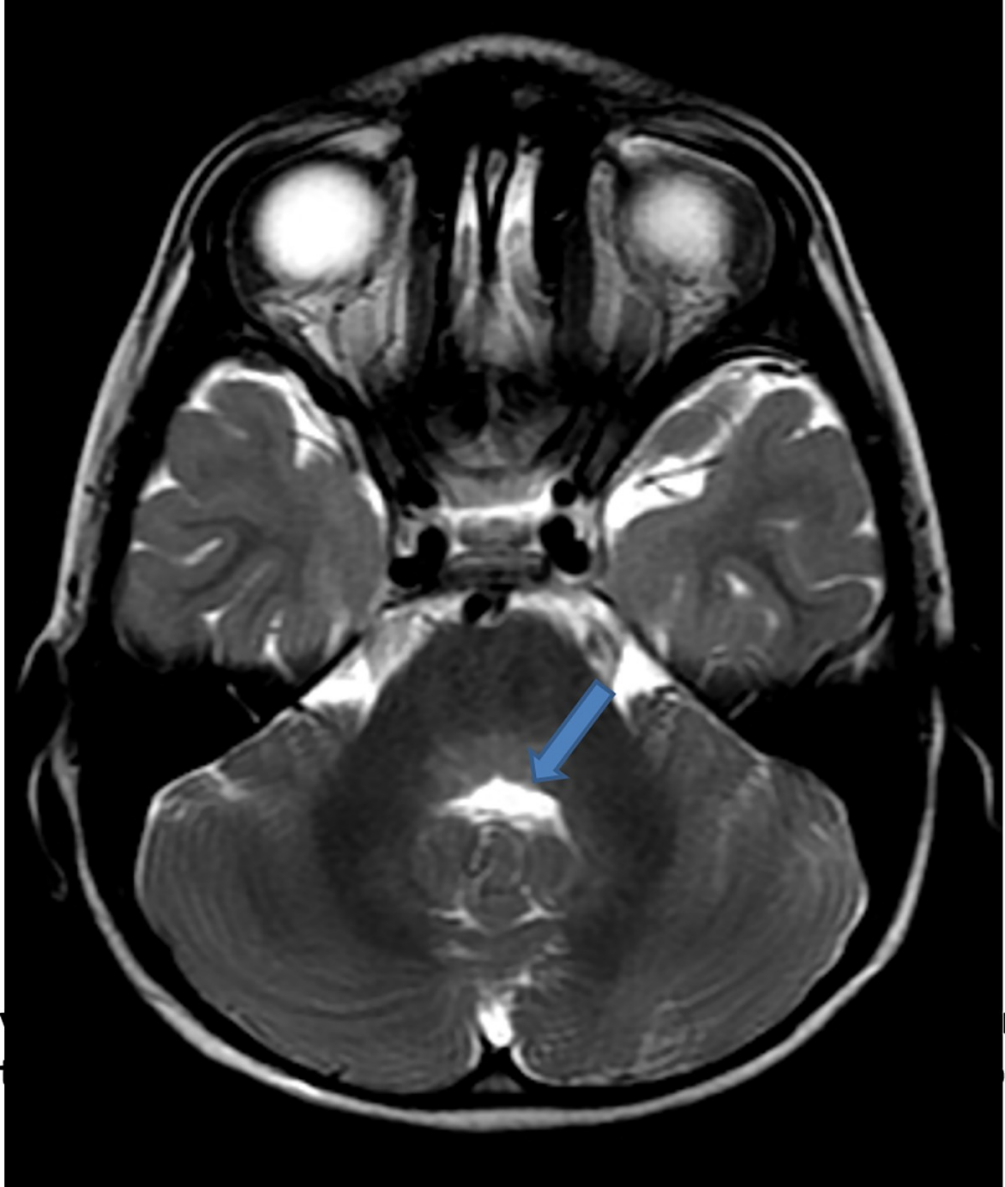
T2 hyperintensities noted in posterior 1/3 of pons. Pontomedullary junction, medulla oblongata, and posterior part of left temporal gyri.

**Fig 4:**
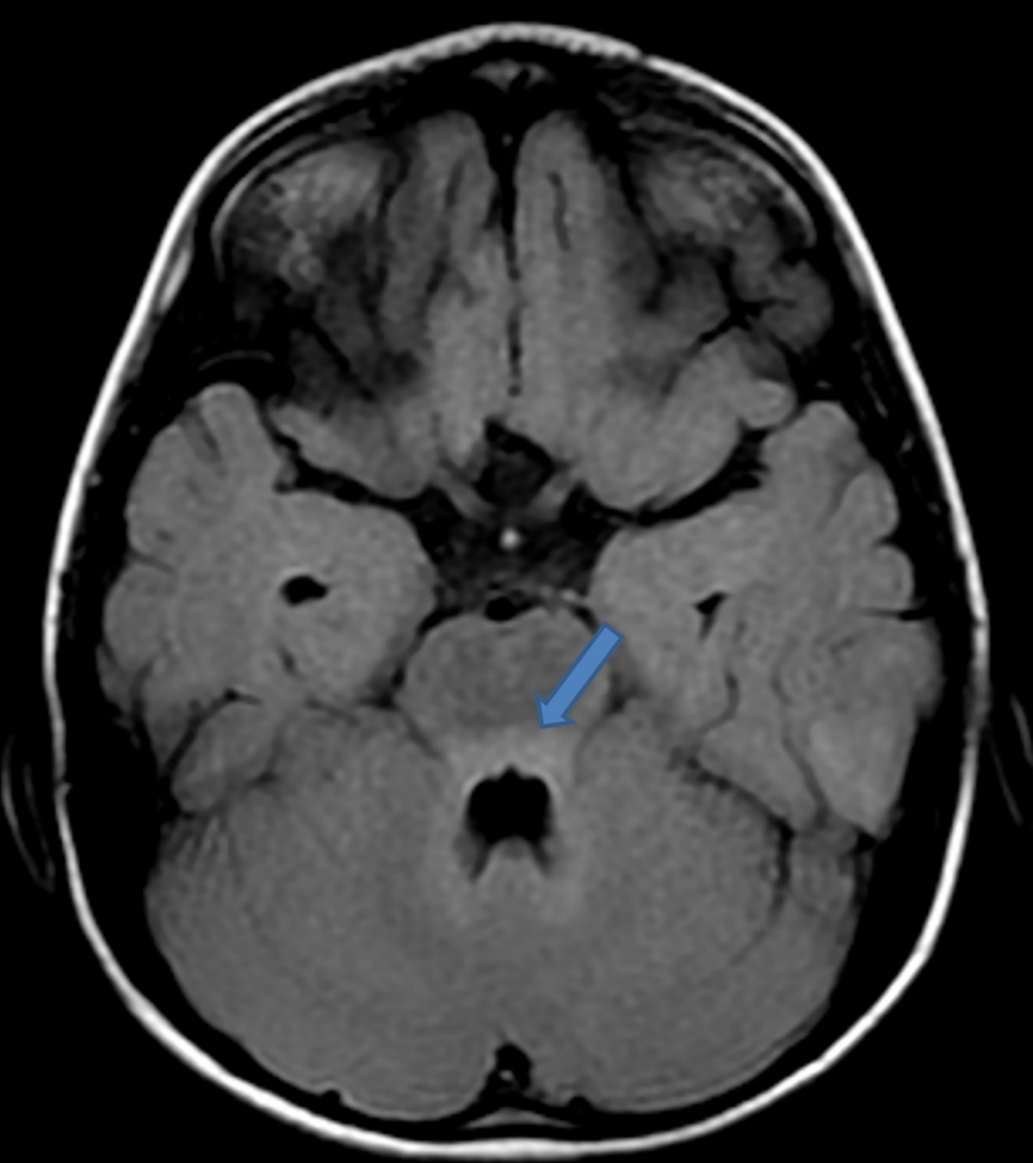
T2/FLAIR hyperintensities noted in posterior 1/3 of pons, pontomedullary junction, medulla oblongata, and posterior part of left temporal gyri.

**Fig 5:**
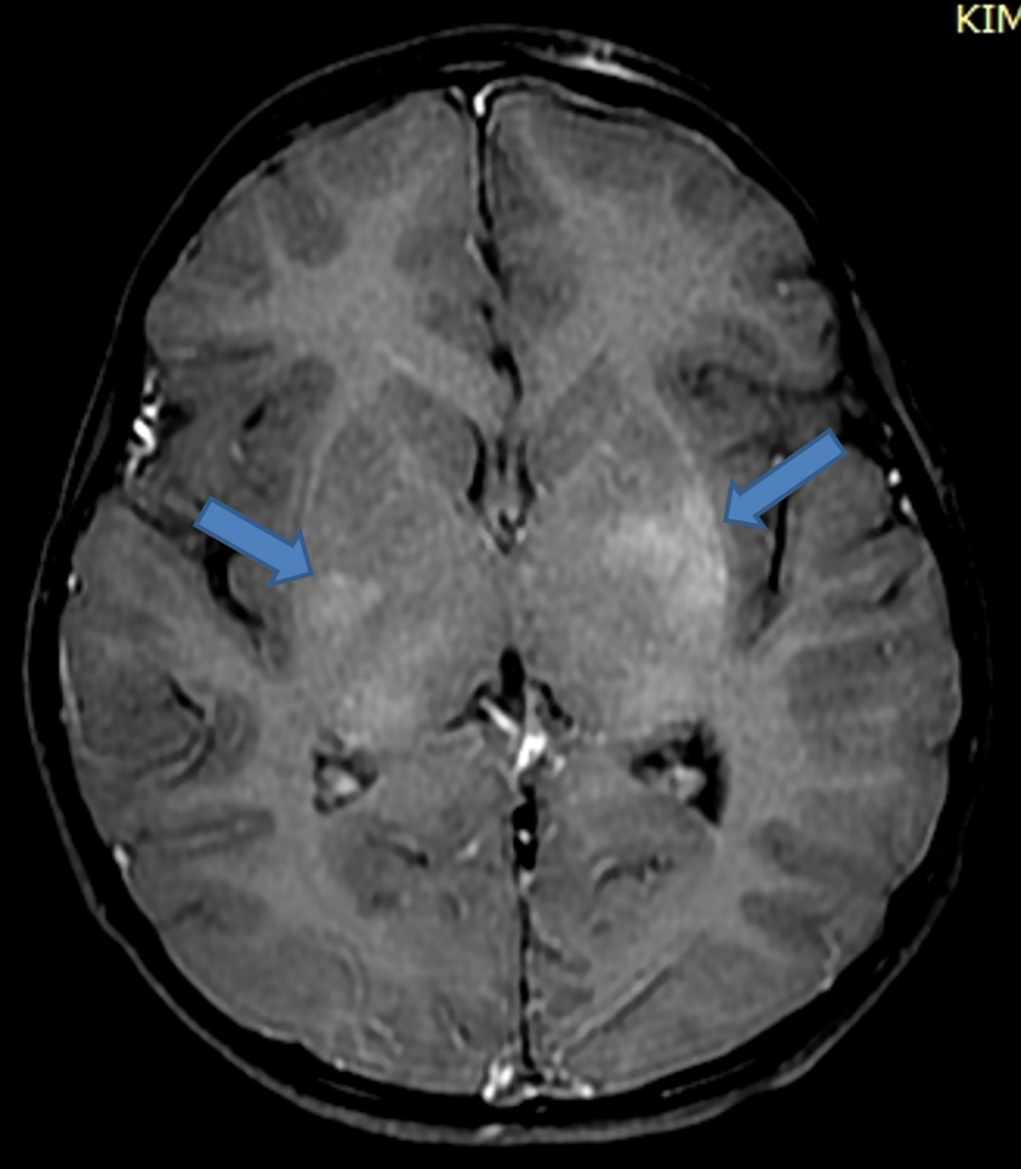
Patchy heterogeneous enhancement seen in bilateral basal ganglia and external capsule on postcontrast study [3].

On day 2 postadmission, saliva and cerebrospinal fluid (CSF) were sent for routine analysis, rabies virus detection through real-time polymerase chain reaction (RT-PCR), and Rapid Fluorescent Focus Inhibition Test (RFFIT) for antibody titre for confirmation of rabies at the department of Neurovirology, NIMHANS, Bengaluru (WHO Collaborating Centre for Reference and Research on Rabies). CSF analysis showed cell count of 25 lymphocytes/μL, proteins 39 mg/dl, glucose 60 mg/dl, chloride 165 meq/l, LDH 16 IU/L, and CSF CRP 0.6 mg/dl. An MRI brain was performed to aid the diagnosis of encephalitis. T2/FLAIR presented with hyperintensities in posterior 1/3 of pons, pontomedullary junction, medulla oblongata, and posterior part of left temporal gyri. Basal ganglia region had no activity suggestive of local inflammation/demyelination. Subsequently on postcontrast study, MRI showed patchy heterogeneous enhancement in bilateral basal ganglia and external capsule. The findings suggested encephalitis probably due to rabies when clinically correlated with history of animal exposure. **(Figs 2–5).**

On day 4, child was intubated and vitals were maintained. The condition of child deteriorated over the next 3 days. Saliva and CSF were negative for rabies viral RNA by real-time PCR. Rabies virus neutralising antibody titre by RFFIT in CSF and serum was reported as <16 UI/mL (antibodies not detected) and 2,048 UI/mL, respectively.

Other causes of encephalitis such as Coronavirus Disease 2019 (COVID-19), Japanese encephalitis, herpes simplex virus, malaria, dengue, and chikungunya were investigated and excluded.

On day 9 postadmission, CSF routine analysis and RFFIT were repeated.

With no progress in child’s health, the parents had consented for extubating the child from ventilator on day 11. The child was extubated at 10.30 AM, and he was declared dead at 11.05 AM. The child died 12 days after the symptoms occurred. CSF report showed cell count of 45 cells/μL (lymphocytes), proteins 48 mg/dl, sugars 112 mg/dl, chloride 123 meq/L, LDH 52 IU/ L, and CRP 0.02 mg/dl. A 4-fold rise in the CSF antibody titre (256 UI/mL) was observed suggestive of rabies encephalitis without any change in the serum values (2,048 UI/mL).

To mainly allay the fear among the health staff, 35 treating doctors including paediatricians, anaesthetists, interns, nurses, and cleaning staff took postexposure prophylaxis along with the family members.

### Ethics statement

Consent from the child’s parents for publication in PLOS journal is taken. The name of the ethical committee is KIMS institutional ethics committee. Approval for the study was granted (KIMS/IEC/103/2020), and confidentiality of the patient was maintained. Also, no specific funding was received for this work.

### Case discussion

The findings in this case suggest that management of wounds in the areas of the head and neck require careful evaluation and treatment with rabies biologicals.

The cause of death in completed rabies postexposure prophylaxis can be attributed to vaccines or RIG not stored in proper cold chain, improper administration of either vaccine or RIG, and inadequate administration of RIG. In the present case, maintenance of cold chain in ice-lined refrigerators (power backup) and temperature monitoring were ensured. No expired vaccine was used, and other patients had received the vaccine from the same batch. This was confirmed by the health centres and patients being alive. The switch in IM and ID route was due to different places of administration of vaccine and not done intentionally. Furthermore, the 2018 expert consensus and the WHO recommendations suggest that switch in route of administration of rabies vaccine also provides the desired protection [3].

In the present case, the wound was on the scalp (thickly innervated with nerves) measuring about 15 cm, and RIG administration was calculated based on body weight (2.7 ml). This quantity of RIG administrated with dilution may not have been sufficient to neutralise the virus in all the exposed parts. However, if the child had received RIG over and above the recommended body weight with or without appropriate dilution, the RIG may have neutralised the virus in all the exposed parts. The Equirab product manual mentions ERIG should not exceed the body weight recommendations because immunoglobulin may partially suppress active production of antibodies [4]. However, Madhusudhana and colleagues had mentioned that calculating the dose of RIG based on body weight may be not necessary [5]. The 2018 Expert Review mentions that with modern biologicals, the risk of systemic reactions to RIG are now considered negligible [3]. This is due to administration limited to and localised in the dermis, systemic distribution might be minimised and not interfere with postvaccine antibody production.

A clinical case was reported in a child who had rabies exposure on the face, received full dose of ERIG into the wound, sutured, 4 doses of ARV (IM) given, and still developed rabies like the present case [6]. One of the causes of rabies prophylaxis failure is that not all wounds were infiltrated adequately with RIG [7], which may be the reason in the present case.

Antemortem confirmation of rabies by a combination of laboratory diagnostic assays (detection of viral RNA in CSF, skin and saliva, and neutralising antibodies in CSF) could be achieved in 40.6% cases similar to the observations of the present case [8].

An MRI of the brain and cervical spine performed using 1.5 Tesla scanner showed bilaterally symmetrical hyperintensities in T2 and FLAIR images in basal ganglia, thalamus, dorsal medulla, and central grey matter of the cervical spinal cord extending to the dorsal segments similar to the present case [9]. Diagnosis of rabies by MRI can be correlated when clinical suspicion is very high.

Postvaccination encephalomyelitis associated with vaccine such as rabies was ruled out in the current case as the CSF titre for rabies antibody showed 4-fold rise through RFFIT [10]. The clinical signs presented are similar to another confirmed rabies death reported in Bengaluru in a paediatric-aged child.[11]

Also, the cause of rabies encephalitis due to suturing an acute wound and in case of direct nerve inoculation of rabies virus has to be considered and beyond the scope of the study.

There is a need for research studies to generate evidence on upper limit or maximum quantity of RIG that can be given in high-risk exposures, along with vaccination for prevention of rabies in India. Moreover, currently, the WHO guidelines recommend local infiltration only, without injecting the remnant of the unused diluted RIG at a distance [12].

Good intensive care with supportive measures may help the occasional patient with rabies encephalitis to survive, but there is an urgent need for novel antivirals and newer therapeutic strategies to improve the outcomes [13].

## Conclusion

Rabies encephalomyelitis, in spite of postexposure rabies prophylaxis, may be attributed to insufficient RIG administration to neutralise the virus locally. There is a need for further studies on the quantity of RIG in high-risk exposures for prevention of rabies.
