# Correction: Rabies encephalitis in a preschool child following postexposure prophylaxis

**DOI:** 10.1371/journal.pntd.0009895

**Published:** 2021-10-25

**Authors:** The PLOS Neglected Tropical Diseases Staff

## Notice of Republication

This article was republished on October 4, 2021, to remove extra files that were not meant to be included at publication. The publisher apologizes for the errors. Please download this article again to view the correct version.
